# Use and exchange of knowledge in the introduction of hospital-based home rehabilitation after a stroke: barriers and facilitators in change management

**DOI:** 10.1186/s12913-022-07618-x

**Published:** 2022-02-17

**Authors:** Margareta Karlsson, Birgitta Nordström

**Affiliations:** 1 Department of Social Sciences, Technology, and Arts, Luleå University of Technology, Luleå, Sweden; 2 Department of Health, Education, and Technology, Luleå University of Technology, Luleå, Sweden

**Keywords:** Collaboration, Knowledge exchange, Improvements, Implementation, Stroke

## Abstract

**Background:**

The purpose of the study was to contribute to research and practice on how the use and exchange of knowledge can facilitate change in health care, specifically methods supporting managers. The study also aimed to investigate barriers related to governance principles that may affect organizational ability to improve quality of care. To achieve the purpose, the study followed a project of hospital-based home rehabilitation after a stroke at a hospital in Norrbotten County, Sweden.

**Methods:**

Seven individual interviews were performed to obtain information from the project members and the managers involved in the project. A group interview with the team and their immediate manager were conducted after the project ended. A thematic analysis was performed to identify and present patterns that formed the results of the study.

**Results:**

The study shows how knowledge was identified, gathered, used, and disseminated in the project. The analysis pointed out how knowledge played an important role from two perspectives: in evidence-based practice in rehabilitation work and for change management. Knowledge exchange and learning across organizational boundaries increased the pace, efficiency, and effectiveness, but collaboration on knowledge, in the sense of joint activities based on a common purpose, only took place within the rehabilitation work. Furthermore, there were indications that governance principles, such as the distribution of financial responsibility and the requirements for official recommendations, influenced the pace of change.

**Conclusions:**

It was shown that the exchange of knowledge and collaboration can facilitate change in health care, but that communication needs to be planned and prioritised. Readiness for change was the basis for the success of the project and for ensuring commitment among those involved. There is also a need for the management to understand how governance principles may affect the efficiency of change work.

**Supplementary Information:**

The online version contains supplementary material available at 10.1186/s12913-022-07618-x.

## Background

As a result of technological development, an aging population, and better treatment of diseases, health care institutions worldwide are calling for change to achieve higher quality and efficiency, and Swedish health care is no exception; however, many such initiatives fail and greater knowledge of both successes and failures is needed [1] Furthermore, there is uncertainty about how the change work has contributed to better care due to a lack of adequate evaluations [2]. There are also requirements that care should be based on evidence-based practice (EBP), meaning that the professionals weigh their own expertise with the best available scientific knowledge and the patient’s situation, experiences, and desires when deciding on treatment and efforts [3]. Despite the increased use of EBP, patients do not always receive the most effective treatments, resulting in a need to reduce the gap between what has proved to be effective and what is actually practised [4]. Research on change and improvements of quality in health care has developed in recent decades in research topics such as Improvement Science [2, 5–7] and Implementation Science [4, 8, 9]. The change work can have different purposes; implementing published evidence where effectiveness is assumed, testing a method or intervention that is known to be effective in other contexts, or initiating new methods or interventions where the effects are unknown [10].

Regardless of purpose, change means that employees need to be involved in the process as change challenges the pursuit of stability [1, 11–13]. To achieve higher quality and efficiency in care, change work, therefore, needs to be a natural part of daily work [5]. This means that change management and learning in the organization come into focus where the goal is to achieve shared understanding and joint action [14]. The identification, gathering, and use of various forms of knowledge, as well as knowledge exchange between employees and organizations, is, therefore, important for efficient change work. Knowledge can be defined as “justified personal beliefs” and be divided into tacit or explicit knowledge. Another way to distinguish knowledge is about “know what”, “know how”, and “know why”. Knowledge would be seen as an asset for the organization and should therefore be managed efficiently to support change, decision-making, and learning [15]. The use of knowledge would also be seen as a two-way process between relevant stakeholders aimed at translating knowledge into action [16].

To increase the probability that the change work will be successful and lead to higher quality for the patients, knowledge of how to achieve organizational readiness for change is essential, i.e., a combination of shared determination and belief in the ability to effect change. The higher the level of readiness, the more likely new initiatives will be taken [11]. Taking employees’ own initiatives into account can increase the possibility of a successful change [12]. To ensure that the actors concerned will participate, three interdependent conditions must be met: expectation, motivation, and commitment [13]. In summary, change is thus facilitated if the actors have the opportunity to influence the change, feel prepared for it, and understand the benefits for the patients [1].

However, change management in the public sector has been affected by governance principles derived from the private sector [17], as well as those that emphasise hierarchy and formal authority [18]. But these principles have been questioned for inconsistencies [19], for example, performance measurement focusing on quantifiable indicators can mean that non-quantifiable aspect of care is ignored, as the saying “what gets measured gets done.” Another example is that the decentralisation of responsibility for finance and operations has increased the requirements for the accountability of managers [20, 21]. The use of a business-like discourse and profit centres within the organization may conflict with the pursuit of collaboration in processes [22]. On the other hand, a structured approach for how to initiate and manage change can give managers a sense of control [23] and experts can support the process of change [24–26]. Introducing processes and methods that have proven effective elsewhere can also facilitate change [9, 27]. Additionally, access to knowledge of outcomes of patient care and improvements is a valuable source for learning [5, 6, 28, 29].

Furthermore, managers need to know how to promote collaboration between actors and organizations to facilitate the change work and the exchange of knowledge [30–32]. As several organizations and professions often are involved in the chain of care, there is a need for coordinating knowledge exchange to reduce the fragmentation for patients with the need for higher levels of care [12, 33]. By identifying something that can act as a bridge in handovers between actors and organizations (a boundary object), coordination and knowledge exchange in different action nets can be facilitated [21, 34, 35]. The sharing of knowledge in an interprofessional team is assumed to increase the quality for patients [36], but different professional logics can hinder this endeavour, for example, if one profession’s values and priorities dominate the collaboration in the team [37]. There is also a risk that team members feel torn between the team and the workplace, while, as time goes on, a team tends to become more autonomous [38].

### Early supported discharge and rehabilitation at home after a stroke

It is well known that stroke is one of the major causes for disability and mortality in Sweden, as it is in most industrial countries [39, 40]. Strokes are characterised by a sudden start and often followed by limitations in the performance of daily living activities [41]. The view of rehabilitation has been expanded to find strategies for how individuals solve tasks in a specific environment [42] and suggests that interventions should shift towards more home rehabilitation [43]. The patient’s perspective of home rehabilitation after a stroke shows that early home rehabilitation in a sparsely populated area influences the person’s ability to return to the life they lived before the stroke [44].

The Swedish Agency for Health Technology Assessment and Assessment of Social Services (SBU) concluded that early supported discharge and rehabilitation at home with an interprofessional team is recommended for patients with minor to moderate symptoms after a stroke because it typically results in fewer deaths and reduced care time and long-term dependence on assistance [45]. The National Board of Health and Welfare (SoS) in Sweden establishes national guidelines for diseases and conditions that affect many people and requires significant resources. The purpose is to support decision-makers in resource allocation. The new guidelines recommended people with mild to moderate symptoms after a stroke to have their rehabilitation at home, either performed by a rehabilitation team from the hospital (prioritised) or by individual care providers from the municipality or primary care [46]. National quality registers with information on medical interventions, procedures, and outcomes contribute to change work and follow-up evaluations of national guidelines [29]. One of the registries is the National Quality Registry for Stroke [47].

Considering that stroke is one of the most common diseases in the world and hospital-based home rehabilitation is prioritised in the Swedish national guidelines, studying the process of introducing the new guidelines from a management perspective can facilitate change management. Following a project from start to end can also increase the understanding of the use of knowledge and collaboration in different phases of the change work. In parallel with this study, an interview study was conducted of how the patients experienced the rehabilitation at home [44].

### Research purpose

The purpose of the study is to contribute to research and practice on how the use and exchange of knowledge can facilitate change work in health care, specifically methods supporting managers. The study also aims to investigate barriers related to governance principles that may affect organizational ability to improve quality of care.

## Methods

### Research setting

The project took place at one of five hospitals in Norrbotten County in Sweden. The hospital covered four municipalities with about 33,000 inhabitants in 2019 and provided health care to persons with heart and geriatric diseases or who recently suffered a stroke. The project involved two of the municipalities and the purpose was to test stroke rehabilitation at home by a hospital-based rehabilitation team. The project aimed to test an organization for the rehabilitation team and to develop procedures for the rehabilitation work and collaboration with actors involved in the chain of care.

The project was carried out from September 2017 to August 2018 and was planned in the spring of 2017 alongside regular work. After the project period, home rehabilitation after a stroke was introduced in the regular operations that covered all four municipalities. The rehabilitation team consisted of a physiotherapist, an occupational therapist, a hospital social worker, and a nurse; two of these individuals applied to be a member of the team, while the other two were assigned to the team because of their current work with stroke patients. Their immediate manager served as the project manager and a physician acted as a medical expert.

### Participants and data collection

A qualitative approach was decided. Individual interviews were performed to obtain information from the individuals involved in the project. A group interview with the rehabilitation team and their immediate manager was chosen to complement the individual interviews and create a dynamic interaction between the participants to capture common aspects of the change work. The rehabilitation team of four persons had between 7 and 18 years of experience in their profession and the three managers had between 8 and 25 years of experience as a manager. For the ease of the reader, the term ‘respondent’ is used when statements come from an individual interview, and ‘participant’ is used when the statements refer to the group interview.

The individual interviews were performed in the spring of 2018 and the group interview in the fall of 2019. All individual interviews were conducted by the first author, while both authors were present in the group interview. The schedule and purpose of the project were discussed with and verified by the immediate manager several times. The individual interviews were semi-structured and lasted between 25 to 85 min, while the group interview lasted approximately 120 min. The interviews were audio-recorded and transcribed verbatim.

The individual interviews were divided into two parts (see interview guide in the appendix in Supplementary material). The first part concerned the project with questions inspired by the framework known as the Knowledge-to-Action process, which comprises activities to identify and use knowledge and aims to facilitate the exchange of knowledge between relevant stakeholders leading to action [16]. The second part of the interviews concentrated on the use of knowledge in general. The interviews with the managers followed the same structure but the dialogue in Part 1 was focused on project management. The group interview covered the same topic as the individual interviews in Part 1.

### Analysis

Thematic analysis was used for the identification, analysis, and presentation of patterns within the collected data [48]. The data set includes all instances in the interviews that concern the project (Part 1) and refer to the topic of knowledge use. The Knowledge-to-Action process comprises activities for identifying and using knowledge [16], which determined the choice of labels for the themes. As a start, the transcripts were read to acquire an overall sense of the content and find examples of the respondent’s views on knowledge. The analysis was conducted in four steps, see Table 1. The first step intended to sort the statements into the two predefined themes: identifying sources of knowledge and using the knowledge. In step 2, the statements were read and analysed without predefined headings to distinguish which sources of knowledge were mentioned. Step 3 intended to determine when the knowledge was identified and used. The Knowledge-to-Action framework [16] emphasize the importance of knowledge exchange between relevant stakeholders, which determined step 4 in the analysis. The analysis process was repeated several times before the final analysis matrix was determined. An example from the analysis can be seen in Table 2.

**Table 1:** The analysis process and results of the analysis

Steps in the analysis process	Results of the analysis	
Step 1: Sort the statements into two predefined themes.	Identifying sources of knowledge	Using the knowledge
Step 2: Distinguish which sources of knowledge the respondents referred to.	Professional knowledge and experiences Results of the rehabilitation and patient experiences Official documents and national statistics Contextual documents Other hospitals procedures and experiences	
Step 3: Determine when the knowledge was gained.	Before project start During the project period After project end	
Step 4: Search for signs of exchange of knowledge.	Within the rehabilitation work For managing the project With external parties

**Table 2:** Example from the analysis of the individual interviews

Statement:	“We could see that in their home, it was completely different, and that the patient managed it [the rehabilitation] better than in the hospital”.
Step 1: Sort the statements into two predefined themes.	Identifying sources of knowledge
Step 2: Distinguish which sources of knowledge the respondents referred to.	Professional experiences
Step 3: Determine when the knowledge was gained.	Before project start
Step 4: Search for signs of exchange of knowledge.	Within the rehabilitation work (with patient)

A summary analysis was conducted on how the knowledge required for the rehabilitation work was linked to the need for knowledge in change work. The group interview intended to confirm the interpretation of data from the individual interviews. First, the participants concluded what happened after the individual interviews. Thereafter, the first author presented the interpretation of the interviews, followed by questions to the participants. The second author presented the interview study with stroke patients [44], which in turn led to a deeper discussion about knowledge use in evidence-based practice.

To ensure the trustworthiness of data, the authors repeatedly discussed the interview guide and the interpretation and analysis of the interviews. The first author made the initial analysis, and, in several meetings with the second author, the analysis was discussed and gradually improved until a consensus was reached. In addition, the first author was well acquainted with research on quality and change work, and the second author was well versed in research on health care processes. Both authors had extensive experience working in administrative and senior positions, as well as in project management.

## Results

Respondents in the individual interviews described, through illustrative examples, of how they identified, gathered, used, and disseminated knowledge before, during, and after the project period. The sources of knowledge mentioned in the statements were professional knowledge, both tacit and explicit, official documents such as the national guidelines and nationwide statistics, contextual documents such as procedures, budget, and follow-up, individual and summarized results of the rehabilitation and patient experiences, and knowledge obtained from actors at other hospitals. The statements also show that the use of knowledge can be described based on the questions why, what, and how.

Furthermore, the respondents’ statements revealed several examples of how they collaborated within the team, with colleagues, with the patient, and with external actors. While the statements of the team members and the managers mostly agreed with one another, the differences were visible in the details: the managers’ statements focused on planning and following up, while the team members’ statements described their roles in the team and the patient work in-depth. The group interview illuminated what happened after the project ended.

The overall analysis of the interviews is presented under four headings that represent different perspectives on how knowledge was handled: identifying sources of knowledge, gathering the identified knowledge, using the knowledge, and collaborating on knowledge. Representative examples of quotes illustrate the analysis.

### Identifying sources of knowledge

A large part of the respondents’ statements focused on what knowledge they identified as important. All respondents were clear about the purpose and each person explained why the project was initiated using almost the exact same wording. A team member said: “We could see that in their home, it was completely different, and that the patient managed it [the rehabilitation] better than in the hospital.”

Statistics was an important impetus to the project. The respondents referred to follow-ups that showed that the population in the hospital area had a greater proportion of cardiovascular diseases than other hospitals. The respondents also mentioned that the hospital had shown good results in the quality register for stroke, but there had been shortcomings in the rehabilitation after discharge. The respondents highlighted that the management’s decision of how to organize home rehabilitation was based on the SBU report and the preliminary national guidelines. The first attempt to get permission to start a project about a year before the actual start was denied by the top management, partly due to a lack of funding; therefore, knowledge of budget and staffing was crucial before deciding on the organization. However, the postponed start was considered positive, as the project could start at a more appropriate time for the hospital without considering external stakeholder requirements.

The need for new procedures was expressed. Information from other hospitals working with home rehabilitation, together with local procedures, was identified as a starting point for administrative procedures, including assessment tools. In response to why home rehabilitation had not started earlier despite successful attempts from other hospitals, respondents said that old habits were difficult to change and that there were no official recommendations. However, the participants in the group interview thought that the project would have started soon anyway because the preparations had been going on for a long time.

### Gathering the identified knowledge

The identified knowledge was gathered both before and during the project period and continued after the project ended. Team members’ own experiences can be described as tacit knowledge visible in the examples from everyday life before, during, and after the project period. The main knowledge sources for the decision to start the project were the report from SBU and the preliminary national guidelines. Another important knowledge source was a regional conference on geriatric care. As it was common knowledge that home rehabilitation had been performed for a long time at a hospital in the nearby region, employees from the hospital were invited to present their organization and procedures.

After the decision to start the project, team members visited a hospital to observe colleagues conducting the rehabilitation work. Team members also came in contact with another hospital and had an e-meeting with them. All documents received were adapted to local conditions by the team. When asked how they validated the information, the members clarified that they copied, tested, and changed the procedures as needed. Team meetings were held regularly to share experiences of the rehabilitation work and improve procedures. An important procedure was the use of assessment tools, and the results were compiled at the end of the project and presented to the management. A manager stated: “It is probably very important that we continue to follow up and measure and think about what we do.”

The team members’ work with the patients was based on their knowledge and experience of rehabilitation at the hospital and the rehabilitation, which was considered essentially the same when performed at home. To update personal knowledge of stroke, a skills improvement course, as well as opportunities to work with more experienced colleagues, were offered. In the group interview, participants expressed a need for increased opportunities to continue education, participate in temporary work at other hospitals, and search for new knowledge for inspiration.

### Using the knowledge

The identified and gathered knowledge, as well as the tacit knowledge and the team members’ previous experience of rehabilitation, were used to manage the project and to share the knowledge with relevant actors. The respondents stated that the patient’s knowledge of their own situation and their goals for recovery were considered essential knowledge when planning the rehabilitation. This knowledge was transferred to the home environment and resulted in a rehabilitation plan. The individual assessment and follow-up during and after the rehabilitation period was used to plan the patient’s further rehabilitation and to improve the procedures. The respondents were pleased with the outcomes, which had reinforced their perception that rehabilitation at home was beneficial for the patients, but as a team member said, “when we started, the idea that patients would be discharged earlier was difficult to communicate with other professionals [involved in the care of the patient].”

On a question regarding the future, the team members stated that they wanted the team to continue and that the knowledge gained was important to preserve and disseminate, but the interviewed managers were vague in their comments and only referred to finances. In the group interview, it was mentioned that the reported outcomes were an important impetus for the management’s decision to introduce the method in regular work.

Dissemination of knowledge before, during, and after the project period was considered very important. Information was provided to hospital staff and employees in the municipalities on several occasions. However, the team members were self-critical, explaining that they could have devoted even more time to information activities before the project started to avoid misunderstandings and opposition, but were unable to do so partly due to the tight schedule. A team member thus recommended the following to other hospitals: “Inform everyone affected or who may be affected. Have information material for different target groups.”

Furthermore, the respondents were uncertain about the interest from other hospitals. As a manager said, “I wish they were a little more curious about what we are doing.” However, after the top management decided to introduce home rehabilitation at the other hospitals, the team members’ experiences were requested. Receiving the improvement scholarship also contributed to the increased attention. The respondents stated that the information they received from other hospitals was crucial in the planning phase, but they admitted that they did not repay the service.

In the group interview, participants were asked how the outcomes of the project could benefit evidence-based practice and person-centred rehabilitation in general, but they said they had not reflected on it. However, they believed the research projects were interesting. Participants were also asked if patients could be involved in improvement work, and they deemed it an interesting idea.

### Collaborating on knowledge

The analysis of signs of collaboration revealed that it was obvious that many professionals were involved either in the project or in the rehabilitation work. The respondents described how they exchanged knowledge through contacts within the project, within the rehabilitation work, and with other partners.

#### Within the rehabilitation work

The patient was considered to be the most important person to collaborate with in order to achieve the patient’s goals. A manager reflected on this power relation in the interview, explaining that “You are the manager in your own home.” The respondents stated that because the hospital was small with short decision paths, collaboration with employees and managers in different departments and with the various health centres and municipalities was uncomplicated. A team member said: “I think it is easier to understand each other and use each other’s skills in a small hospital.” However, a need for improved collaboration on the patient’s rehabilitation plan before discharge was also mentioned*.*

#### For managing the project

The respondents were asked which persons they considered most important for the realisation of the project. The team mentioned the managers, while the managers’ opinion appeared in the following quote: “It was the commitment and enthusiasm of the staff that made it possible to start.” One factor for success mentioned by the managers was that the team members were handpicked based on their expressed interest. The managers pronounced the importance of involving the employees early on in change work, but since the top management rejected the project the first time, the employees’ commitment was put on hold.

The team members showed a great commitment to the work in the team and explained how the procedures and weekly meetings were crucial for knowledge sharing. As a team member mentioned: “We have a team meeting every week, but that is not true, we meet almost every day.” Over time, the respondents stated that their individual experiences had become common knowledge, which facilitated collaboration and the opportunity to replace each other when needed.

A physician acted as a medical expert to the team and administrative employees were consulted to some extent, but specially appointed facilitators did not participate in the project. One reason for this lack of participation was due to feelings of doubt about what skills the experts could provide. However, the collaboration with scholars was considered important to gain knowledge about evidence-based methods for evaluation. The immediate manager acted as a project manager and the other two managers were regularly informed of the project’s progress.

#### With external parties

Especially important were the contacts with another hospital’s employees who were already working with home rehabilitation, mediated by a former employee. Other information activities concerned, for example, actors in municipalities and politicians. A desire to collaborate with the other hospitals in the region was expressed. As a team member said: “Twice a year, all hospital social workers in the region meet in joint working groups. There you have the opportunity to talk about this [stroke rehabilitation].” The respondents also expressed the idea that knowledge gained could be translated to other hospitals and other patient groups.

### Sources of knowledge and their use in health care change work

The project aimed to test how to organize the rehabilitation team and develop new procedures for the rehabilitation in the patient’s home. The analysis of the interviews showed that knowledge use played an important role in achieving the aim: (1) in evidence-based practice in the rehabilitation work, and (2) in change management when introducing the new method.

#### Evidence-based practice

The interviews showed that the care of the patients was based on a deep knowledge of what researchers consider to be effective rehabilitation after a stroke and based on the national guidelines, professional experiences of stroke rehabilitation, and the patient’s own knowledge and goals; that is, an evidence-based practice. Available resources provided the framework for the organization of rehabilitation. An assessment of the patient’s recovery provided knowledge for improvements in the rehabilitation work. The outcomes of the patient group gave impetus to future improvement efforts.

#### Change management

**Fig. 1:**
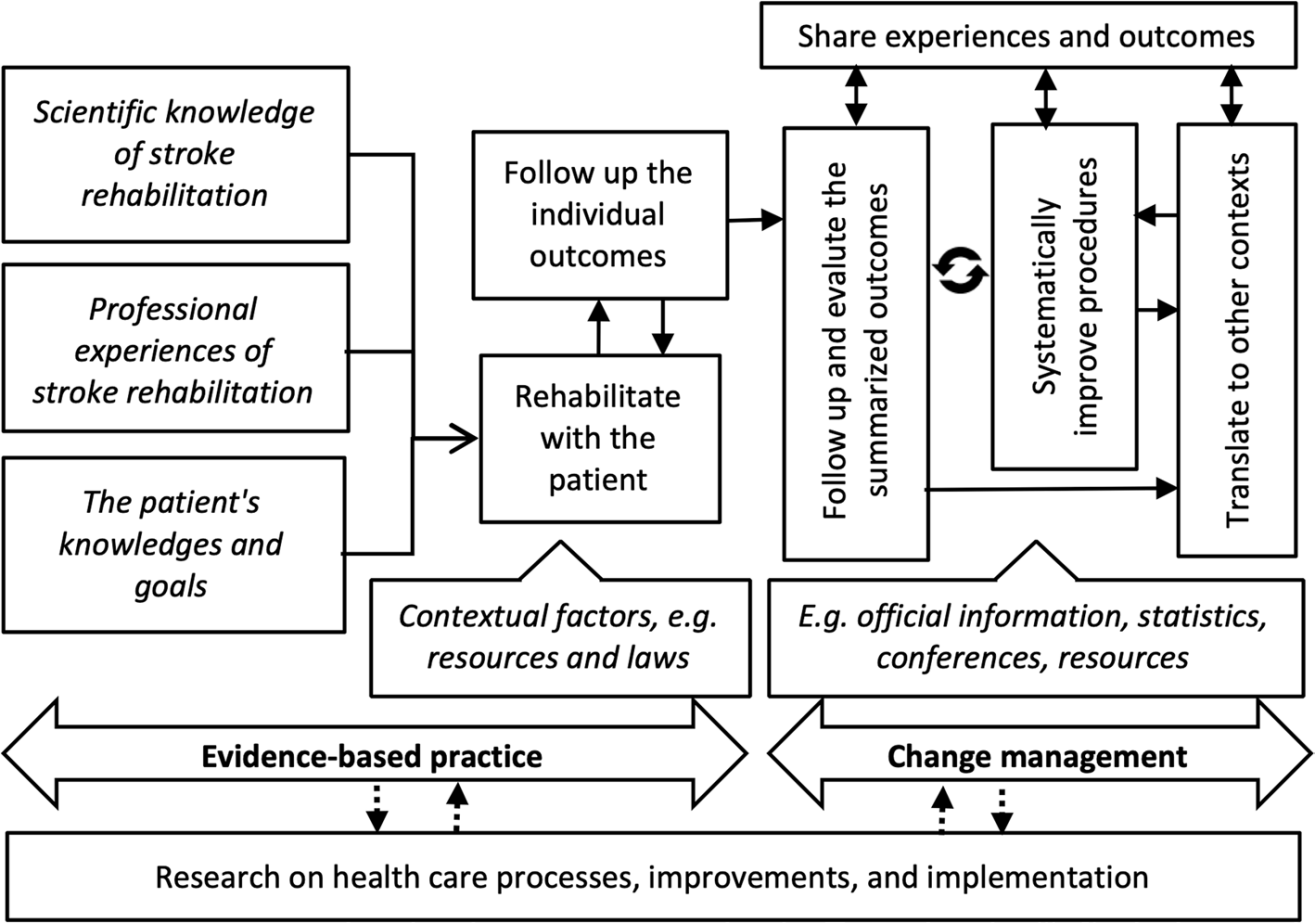
Sources of knowledge used in the project

The analysis also showed how knowledge was identified, gathered, used, and disseminated in the project. The knowledge used for project management were contextual such as statistics, procedures, and resources, official documents, and information from other hospitals. The follow-up procedures and outcomes became important for the decision-makers and for disseminating knowledge to various stakeholders. After the project period, systematic improvement work was introduced. Thoughts of how to apply the knowledge gained to other contexts were expressed but not yet implemented. Figure 1 summarises how different sources of knowledge were used in the change work.

## Discussion

The study shows that the identification, gathering, use, and dissemination of knowledge took place in two mutually dependent parts of the change work — (1) the use of evidence-based practice in rehabilitation for stroke patients and (2) the management of the project — but it also shows that the link between the two parts was quite weak. This emphasizes that capacity and capability for change need to be built at all levels in the organization and points out the need for systematic collection of knowledge about care outcomes in daily work.

To facilitate change, management needs to have knowledge of organizational readiness for change [1, 11]. To investigate these conditions among the actors, we applied the conditions for creating organizational action: expectation, motivation, and commitment [13]. Expectations for the introduction of the new method were built up over a long period of time through external contacts. The official documents confirmed the scientific basis, provided guidance for management decisions both locally and centrally, and raised further expectations among actors. The appointment of team members according to their stated interest or current work increased motivation. Mutual commitment and confidence in the team’s ability to manage the project developed as the team became more autonomous. Disseminating experiences and receiving the improvement scholarship contributed to the enthusiasm. In summary, all the prerequisites for success were found among those directly involved in the project. However, a readiness for change was not obvious among the other actors affected, which may be attributed to the fact that old habits can be difficult to change, as one manager said.

In addition, the study shows that barriers due to governance principles may affect the pace of change. The disaggregation of units and decentralisation of financial accountability may delay change and hindering collaboration in processes and knowledge exchange [12, 19–22]. However, since the project was carried out within a unit, the project manager did not have to negotiate resources with other managers, but this also pointed out the problem of budgeting rules within the hospital. Hesitancy among top management due to no demands from authorities indicate insufficient readiness for change which can delay changes even if there is knowledge of applications at other hospitals [11, 18, 19]. Information about outcomes was crucial for politicians, top management, and other actors affected. Early planning for a structured collection of patient care outcomes and learning about improvements in one’s own and other organizations would therefore facilitate future change work [2, 5, 7, 9, 27–29].

The study showed that the exchange of knowledge and learning across organizational boundaries increased the pace, efficiency, and effectiveness of the change work [30–32]. But collaboration on knowledge, in the sense of joint activities based on a common purpose, only took place within the rehabilitation process. The exchange of knowledge related to the project was not mutual and mostly took place through temporary informal contacts. The support of experts can contribute to improved quality of the change work, but the choice of organization (e.g., in central staff or support function) may hinder efficient use [21, 25, 26]. Formalised and planned collaboration in networks and projects can both legitimise and promote knowledge exchange [22, 38]. Strategically organized and structured procedures for change management and knowledge exchange, supported by research, would also benefit managers [1, 4–7, 16, 23].

Continuous communication within the team contributed with tacit knowledge and ensured a common perspective in the rehabilitation work [36, 37]. Dividing working time between the team and other tasks, facilitated communication with colleagues and reduced the risk of the team becoming detached while maintaining a professional identity, but it can also be perceived as stressful for the employee [37, 38]. A need for support of the exchange of knowledge in handovers over organizational boundaries was pronounced. Creating a plan that includes rehabilitation throughout the chain of care can thus facilitate collaboration and coordination between actors, organizations, and the patient, thereby avoiding disruptions in rehabilitation [12, 21, 34, 35].

### Strengths and limitations

A mayor limitation was that the study was small and examined a simple change project. This limitation influenced the choice of methods for collecting and analysing data to strengthen the findings. The study included only seven participants, but information with sufficient power is more important than the size of the sample [49]. Accordingly, all the directly involved persons in the project were interviewed. The respondents’ experiences due to their profession, as well as our choice of semi-structured interviews that challenged the respondents’ beliefs, were expected to reduce the limitation and contribute to the relevance of the study. The main strength of the study was the participants’ view of the project in their own words and that the statements correspond to the other participants’ statements. Another strength was that a model for the use and exchange of knowledge inspired the interview guide and the analysis process [16]. Situational factors such as the characteristics of the participants, the project, and the context were described. The analysis process was validated with the criteria proposed by Braun and Clark [48] and is described in steps to help the reader to evaluate the results. To further strengthen the findings, participants were invited to a meeting for participant validation [50]. The authors’ acquaintance with qualitative research and their different experiences of research and practice contributed to a heterogeneous perspective on change management in health care. To facilitate transferability, a description of the context was provided. Furthermore, the authors had no relation to the specific project and participants.

## Conclusion

The study followed a project of introducing hospital-based home rehabilitation after a stroke. The study shows how knowledge was identified, gathered, used, and disseminated in the project. The study intended to contribute to research and practice regarding methods in supporting managers. Although the project can be considered a success, some lessons might be considered for future projects:

- *Repeatedly ensure that information is received and understood by all actors affected by the change.* Although the hospital was small with both informal and formal communication channels, respondents said they did not provide enough information to ensure readiness for change among other actors in the chain of care.
- *Plan for a systematic gathering of knowledge about patient care outcomes and results of change work, including both failures and successes.* Reliable information was an important basis for the decision on introducing the method at other hospitals in the region. However, knowledge of how to measure the efficiency of the specific change was lacking, which could impede other changes that may contribute to lower costs.
- *Facilitate for change agents to use expert knowledge.* Personal knowledge of expert competence was lacking and contributed to a lower demand for support.
- *Facilitate collaboration on knowledge in networks and projects (*e.g.*, within a profession and between interprofessional teams).* Informal temporary contacts for knowledge exchange and the dissemination of outcomes placed great demands on team members to know how to find relevant channels.

In addition, there were indications in the interviews of some issues related to governance principles that may affect change management. Demands for financial responsibility and scientific evidence delayed the introduction of the new rehabilitation method, which indicates that management should be aware that governance principles can affect the possibility of achieving higher quality and efficiency [19]. The study also showed that there was an organizational readiness for change among the actors directly involved before the project was decided, but not among the top management. This indicates the importance of being ready for change at all organizational levels. Building up expectations about improvements that are not realised can contribute to reduced motivation and commitment, thus affecting future change work.

Furthermore, a greater understanding is needed of how the exchange of knowledge can facilitate change and how governance principles might counteract this effort. This could be achieved through increased collaboration between different research themes and with practitioners in health care.

## Supplementary Information

**Additional file 1.** Interview guide.

## Data Availability

The qualitative data for this study is safely stored at Luleå University of Technology, Sweden. The datasets used and analysed during the current study are available from the corresponding author on reasonable request.

## References

[1] Nilsen P, Seing I, Ericsson C, Birken SA, Schildmeijer K (2020). Characteristics of successful changes in health care organizations: an interview study with physicians, registered nurses and assistant nurses. BMC Health Serv Res.

[2] Hill JE, Stephani A, Sapple P, Clegg AJ (2020). The effectiveness of continuous quality improvement for developing professional practice and improving health care outcomes: a systematic review. Implement Sci.

[3] Sackett DL, Rosenberg WM, Gray JA, Haynes RB, Richardson WS (1996). Evidence based medicine: what it is and what it isn’t. BMJ.

[4] Westerlund A, Nilsen P, Sundberg L (2019). Implementation of implementation science knowledge: the research-practice gap paradox. Worldv Evid Based Nu.

[5] Batalden PB, Davidoff F (2007). What is “quality improvement” and how can it transform healthcare?. Qual Saf Health Care.

[6] Marshall M, Pronovost P, Dixon-Woods M (2013). Promotion of improvement as a science. Lancet.

[7] Bergman B, Hellström A, Lifvergren S, Gustavsson SM (2015). An emerging science of improvement in health care. Qual Eng.

[8] Eccles MP, Mittman BS (2006). Welcome to implementation science. Implement Sci.

[9] Nilsen P, Ståhl C, Roback K, Cairney P (2013). Never the twain shall meet? A comparison of implementation science and policy implementation research. Implement Sci.

[10] Fischbacher CM, Lewsey J, Muirie J, McCartney G. A critical reflection on the use of improvement science approaches in public health. Scand J Public Health. 2021:1–6. 10.1177/1403494821990245 33596733

[11] Weiner BJ (2009). A theory of organizational readiness for change. Implement Sci.

[12] Hansson A, Svensson A, Ahlström BH, Larsson LG, Forsman B, Alsén P (2018). Flawed communications: health professionals’ experience of collaboration in the care of frail elderly patients. Scand J Public Health.

[13] Brunsson N (2000). The irrational organization: irrationality as a basis for organizational action and change.

[14] Crossan MM, Lane HW, White RE (1999). An organizational learning framework: from intuition to institution. Acad Manag Rev.

[15] King WR, King W (2009). Knowledge management and organizational learning. Knowledge management and organizational learning. Annals of information systems, 4.

[16] Graham ID, Logan J, Harrison MB, Straus SE, Tetroe J, Caswell W (2006). Lost in knowledge translation: time for a map?. J Contin Educ Heal Prof.

[17] Hood C (1991). A public Management for all Seasons?. Public Adm.

[18] Adler PS (2001). Market, hierarchy, and trust: the knowledge economy and the future of capitalism. Organ Sci.

[19] Diefenbach T (2009). New public management in public sector organizations: the dark sides of managerialistic ‘enlightenment’. Public Adm.

[20] Arman R, Liff R, Wikström E (2014). The hierarchization of competing logics in psychiatric care in Sweden. Scand J Manag.

[21] Andersson T, Liff R (2012). Multiprofessional cooperation and accountability pressures: consequences of a post-new public management concept in a new public management context. Public Manag Rev.

[22] Karlsson M, Garvare R, Zingmark K, Nordström B (2020). Organizing for sustainable inter-organizational collaboration in health care processes. J Interprof Care.

[23] Nyström ME, Höög E, Garvare R, Bäck MA, Terris DD, Hansson J (2018). Exploring the potential of a multi-level approach to improve capability for continuous organizational improvement and learning in a Swedish healthcare region. BMC Health Serv Res.

[24] Liff R, Andersson T. Experts’ contribution to strategy when strategy is absent. A case study of quality experts in hospitals. Public Manag Rev. 2020:1–21.

[25] Westerlund A, Garvare R, Höög E, Nyström ME (2015). Facilitating system-wide organizational change in health care. Int J Qual Serv Sci.

[26] Gibbs T (2019). Running on goodwill: the value of co-operative relationships at work. Perspectives: Policy and Practice in Higher Education.

[27] Oborn E, Barrett M, Prince K, Racko G (2013). Balancing exploration and exploitation in transferring research into practice: a comparison of five knowledge translation entity archetypes. Implement Sci.

[28] Höög E, Lysholm J, Garvare R, Weinehall L, Nyström ME (2016). Quality improvement in large healthcare organizations. J Health Organ Manag.

[29] Sparring V, Granström E, Sachs MA, Brommels M, Nyström ME (2018). One size fits none–a qualitative study investigating nine national quality registries’ conditions for use in quality improvement, research, and interaction with patients. BMC Health Serv Res.

[30] Craps M, Vermeesch I, Dewulf A, Sips K, Termeer K, Bouwen R (2019). A relational approach to leadership for multi-actor governance. Adm Sci.

[31] Kennedy J, McKenzie I, Thomas J (2019). Developing effective collaborations: learning from our practice. Adm Sci.

[32] Schruijer SG (2020). Developing collaborative interorganizational relationships: an action research approach. Team Perform Manage.

[33] Eriksson E, Andersson T, Hellström A, Gadolin C, Lifvergren S (2020). Collaborative public management: coordinated value propositions among public service organizations. Public Manag Rev.

[34] Lindberg K, Czarniawska B (2006). Knotting the action net, or organizing between organizations. Scand J Mbmt.

[35] Sullivan H, Williams P (2012). Whose kettle?: exploring the role of objects in managing and mediating the boundaries of integration in health and social care. J Health Organ Manag.

[36] Grol RP, Bosch MC, Hulscher ME, Eccles MP, Wensing M (2007). Planning and studying improvement in patient care: the use of theoretical perspectives. Milbank Q.

[37] Gadolin C, Wikström E (2016). Organising healthcare with multi-professional teams: activity coordination as a logistical flow. SJPA.

[38] Axelsson R, Axelsson SB (2006). Integration and collaboration in public health—a conceptual framework. Int J Health Plann Manag.

[39] Donnan GA, Fisher M, Macleod M, Davis SM (2008). Stroke. Lancet.

[40] SoS (Socialstyrelsen [The National Board of Health and Welfare]). Statistics on Stroke 2018. Stockholm: Socialstyrelsen; 2019. Available from: https://www.socialstyrelsen.se/globalassets/sharepoint-dokument/artikelkatalog/statistik/2019-12-6486.pdf. Accessed in 27 Aug 2021

[41] Veerbeek JM, Kwakkel G, van Wegen EE, Ket JC, Heymans MW (2011). Early prediction of outcome of activities of daily living after stroke: a systematic review. Stroke.

[42] Shumway-Cook A, Woollacott MH (2007). Motor control: translating research into clinical practice.

[43] Hillier S, Inglis-Jassiem G (2010). Rehabilitation for community-dwelling people with stroke: home or Centre based? A systematic review. Int J Stroke.

[44] Nordström B, Kassberg A-C, Axelsson SW (2020). Stroke survivors’ experiences of early person-centered rehabilitation at home – living in sparsely populated areas. J Neurol Neurocrit Care.

[45] SBU (Statens beredning för medicinsk och social utvärdering [The Swedish Agency for Health Technology Assessment and Assessment of Social Services]). Rehabilitation at home after early supported discharge (ESD) for elderly patients after stroke. Stockholm: Statens beredning för medicinsk och social utvärdering (SBU). SBU report no 234 (in Swedish). Summary in English accessed 27 Aug 2021. Available from: https://www.sbu.se/en/publications/sbu-assesses/rehabilitation-at-home-after-early-supported-discharge-esd-for-elderly-patients-after-stroke/

[46] SoS (Socialstyrelsen [the National Board of Health and Welfare]) (2020). Nationella riktlinjer för vård vid stroke.

[47] National Quality Registry for stroke (Riksstroke) (1994). Nationella Kvalitetsregister [National Quality Registries].

[48] Braun V, Clarke V (2006). Using thematic analysis in psychology. Qual Res Psychol.

[49] Malterud K, Siersma VD, Guassora AD (2016). Sample size in qualitative interview studies: guided by information power. Qual Health Res.

[50] Barbour RS (2005). Making sense of focus groups. Med Educ.

