# Supplementary material for: Use and exchange of knowledge in the introduction of hospital-based home rehabilitation after a stroke: barriers and facilitators in change management
Source: BMC Health Serv Res. 2022 Feb 17;22:216. doi: 10.1186/s12913-022-07618-x (PMC8851767; doi:10.1186/s12913-022-07618-x)
Supplement: Supplementary file 1 — Additional file 1. Interview guide. [file 12913_2022_7618_MOESM1_ESM.docx]

## Additional file 1 – Interview guide

The interviews were held in Swedish, and the interview guide was therefore translated into English.

**Personal information**

Name, Position, Workplace, Years in the profession, Year at the current workplace.

***Part 1: About the project***

**Introductory question**

1. Tell us how you got involved in the project. When in time did you join the project? What role do you have in the project?

**Identify the problem and identify, review, select relevant knowledge**

2. How did you perceive the background to the project? What were the problems to be solved?

3. Do you know of other hospitals that have already introduced the method and what were the results?

**Adapt knowledge to local context**

4. Why do you think this project started at this hospital? Are there any specific success factors here?

5. Who have been the driving force behind the project?

**Assess barriers to knowledge use**

6. Were there any obstacles that needed to be overcome before the project could start? What obstacles? How were the obstacles handled?

7. Do you feel that you have the right skills to work with rehabilitation at home? Have you undergone any skills-enhancing activities?

**Select, tailor, implement interventions**

8. What is the basic difference between home-based stroke rehabilitation and stroke rehabilitation in a ward?

9. When it was decided that the project would start. How did you develop the necessary routines? Which people participated?

10. Do you think that these routines are clearly described? Are they known by relevant actors?

11. Have people outside the project acted as support for the team? What support was offered?

**Monitor knowledge use**

12. Are the routines decided at the beginning of the project applied today or have there been changes over time? What types of changes?

**Evaluate outcomes**

13. Have you discussed your patient experiences in the team? Have the other team members raised similar issues?

14. Has the project shown any concrete outcomes on improvements for patients? Which?

15. What consequences have you noticed regarding the business or the staff? What has gotten better? Has anything gotten worse?

**Sustain knowledge use**

16. What do you think will happen when the project ends?

17. How do you want knowledge and experience from the project to be utilized and disseminated to other hospitals?

18. Have you received questions from colleagues from other hospitals about the project?

**Part 2: Knowledge use in health care in general**

19. How do you interpret “evidence-based practice” from your own perspective?

20. What sources of knowledge do you consider to be most important to keep track of within your professional field?

21. New national guidelines or other official documents that affect your work – how do you get access to them?

22. Which actors in the region do you consider to be particularly important for disseminating new knowledge about evidence-based practice? In what way are they important? What can they help you with?

23. How do you get knowledge about how the patients experience the care?

24. How do you reflect on your own experiences from daily work and patient care?

25. Do you share your experiences of with others in your workplace? Can you share their experiences?

26. Do you think that your experiences are used in improvement work at your workplace?

27. Is there anything else you have thought about when it comes to evidence-based practice and how it affects the improvement work?
